# Integrated Design and Experiment of a Micro-Vibration Isolation and Pointing Platform for Large Space Optical Payloads Based on Voice Coil Motors

**DOI:** 10.3390/s25041179

**Published:** 2025-02-14

**Authors:** Yilin Guo, Jian Zhou, Zehao Gao, Bo Feng, Minglong Xu

**Affiliations:** State Key Laboratory for Strength and Vibration of Mechanical Structures, School of Aerospace Engineering, Xi’an Jiaotong University, Xi’an 710049, China; 2201323170@stu.xjtu.edu.cn (Y.G.); gaozehao7268@stu.xjtu.edu.cn (Z.G.); bofeng@xjtu.edu.cn (B.F.)

**Keywords:** integrated isolation and pointing platform, micro-vibration, voice coil motor, large space optical payloads

## Abstract

This paper presents the design of an integrated micro-vibration isolation and pointing platform with a four-leg structure, incorporating pitch and yaw adjustment functions using voice coil motors. The primary objective is to mitigate the impact of spacecraft-generated micro-vibrations on the pointing accuracy and imaging clarity of large space optical payloads while adhering to lightweight requirements. The research methodology encompasses three main phases. Initially, a simplified dynamic model of the integrated platform is established, and dynamic control equations are derived based on the proportional–integral–derivative (PID) control strategy. The effects of centroid deviation and control parameters on the control efficacy are analyzed. Subsequently, a principle prototype of the two-dimensional micro-vibration isolation and pointing platform is designed, detailing the development of the membrane, actuator, legs, and integrated system. Finally, a ground test verification system is implemented under gravity unloading conditions using elastic strings. The experimental results demonstrate the platform’s effective vibration isolation and pointing capabilities, achieving a 23 dB attenuation effect at the fundamental frequency. Furthermore, the PID control algorithm exhibits enhanced isolation performance at low frequencies and facilitates directional tracking of target signals.

## 1. Introduction

In target tracking tasks performed by space optical payloads, precise and stable wide-range pointing adjustments of the optical components are essential. However, micro-vibrations generated by spacecraft components (e.g., flywheels) significantly impact the tracking and aiming accuracy of these optical payloads [1,2,3,4]. To mitigate the effects of micro-vibrations on large space optical payloads and enable precise tracking and pointing, these payloads must be mounted on platforms that offer exceptional stability and extremely high-precision tracking and pointing capabilities.

Vibration isolation technologies for space telescopes are primarily categorized into three main types: active, passive, and active–passive [5,6]. Passive vibration isolation technology offers high performance and stability, effectively suppressing high-frequency vibrations without additional energy input. However, it is less effective in mitigating low-frequency vibrations [7,8,9]. Active vibration isolation technology employs control algorithms to generate corresponding angles and displacements and drive actuators to counteract disturbances, typically used for suppressing low-frequency micro-vibrations [6,10]. Active–passive vibration isolation technology [11,12] combines the advantages of both methods, providing effective isolation for low- and high-frequency vibrations. The Hubble Space Telescope (HST), which carries multiple optical payloads, utilizes passive liquid dampers designed by Honeywell to suppress reaction flywheel vibrations [13]. The James Webb Space Telescope (JWST) isolation system comprises four cross-arranged graphene beams with a constrained damping layer [14]. The Miniature Vibration Isolation System (MVIS) [15] for satellites, sponsored by the United States Air Force Research Laboratory, employs a bipod structure arranged in different positions to form a hexapod architecture for vibration isolation. An active vibration control system named SUITE [16] with a Gough–Stewart configuration is mounted on the PICOsat small satellite designed in the United States, primarily to maintain the on-orbit stability of onboard optical instruments under low-amplitude vibrations or jitters. Lee et al. [7,17] developed hybrid vibration isolators with a hexapod structure and corresponding hybrid vibration control technologies, which demonstrated good isolation performance in on-orbit experiments.

Pointing platforms can be categorized into two configurations: serial and parallel. Serial platforms typically possess greater mass and volume, potentially increasing transportation costs. Furthermore, their serial structures are susceptible to cumulative motion errors [18], and their structural stability is comparatively low. These factors collectively contribute to the reduced pointing accuracy of these platforms. The Gough–Stewart configuration represents the most prevalent parallel pointing platform [19,20], offering high pointing control accuracy, robust fault tolerance, stable and compact structures, and substantial load-bearing capacities. This configuration enables precise, wide-range target tracking for optical terminals on spacecraft. Schwalm et al. [21] investigated the Fourier transform imaging spectrometer and achieved a pointing accuracy within 4″. The JWST’s secondary mirror demands micrometer-level pointing accuracy, necessitating a precision pointing platform with the Gough–Stewart configuration for ground testing [22]. The Hobby–Eberly Telescope employs a primary mirror adjustment scheme, utilizing a six-degree-of-freedom parallel platform as the adjustment mechanism to attain precise tracking and positioning with an accuracy of ±5 μm.

The research above primarily concentrates on platforms with a singular isolation or pointing function. However, integrated platforms for vibration isolation and pointing can simultaneously address both requirements while conserving considerable space and mass. The Spacecraft Research and Design Center at the United States Naval Postgraduate School (NPS) developed a Precision Pointing Hexapod (PPH) platform for the vibration isolation of space optical imaging payloads [23]. This platform employs six driving legs powered by voice coil motors, offering vibration isolation and precision pointing capabilities. Nevertheless, its substantial single-leg suspended mass impacts system linearity, resulting in relatively ineffective vibration isolation for high-frequency disturbances. A collaborative effort between the Hood Technology (HT) company and the University of Washington (UW) created the HT/UW vibration isolation and pointing platform, which achieved large-angle pointing and vibration suppression using a voice coil actuator with an extensive stroke and large air gap [24]. This actuator attains a fundamental frequency of 3 Hz through axial diaphragm spring deflection, with a driving stroke of 10 mm. Additional research and applications concerning integrated vibration isolation and pointing technology have been conducted [25,26,27].

The aforementioned research primarily focuses on relatively lightweight payloads (under 100 kg), with limited investigation into integrated vibration isolation and pointing platforms for large space optical payloads (exceeding 100 kg). Du et al. [28] developed a Stewart structure-based active–passive vibration isolation platform for large space optical loads, utilizing electromagnetic dampers and piezoelectric actuators. They validated the platform’s active vibration isolation performance through simulation analysis. Similarly, Tang et al. [29] constructed an active vibration isolation platform for large space optical loads using the Stewart structure, employing voice coil motors as actuators. Their study confirmed the effectiveness and robustness of fuzzy proportional–integral–derivative (PID) control for active control. Both studies employed a six-legged Stewart configuration platform, resulting in a considerably massive platform.

To mitigate the impact of spacecraft-induced micro-vibrations on the pointing accuracy of large space optical payloads while adhering to lightweight requirements, this paper develops an integrated vibration isolation and pointing platform with a four-leg structure utilizing voice coil motors. This study begins with a theoretical analysis of the designed platform, followed by the creation of a principle prototype, which includes the design of the membrane, actuators, legs, and the integrated platform. Subsequently, the platform undergoes testing to validate the feasibility of its structural design and control system.

## 2. Theoretical Analysis

### 2.1. Basic Principle

**Figure 1:**
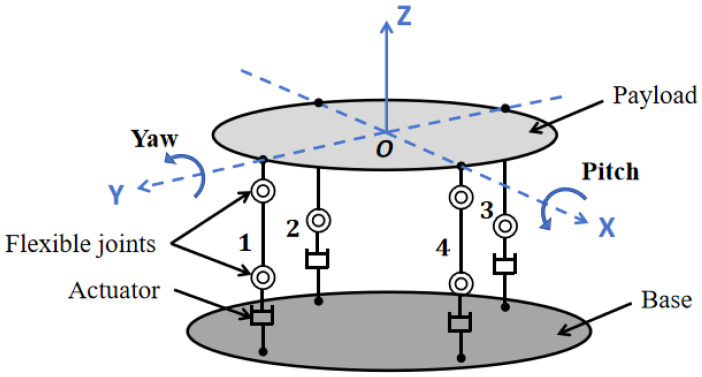
Schematic diagram of the integrated platform.

The structural schematic diagram of the integrated vibration isolation and pointing platform is illustrated in Figure 1. The platform primarily comprises a payload, four legs with vibration isolation and pointing capabilities, and a base. Each leg incorporates an actuator, two flexible joints, and connecting components. To achieve decoupling in the pitch and yaw directions, the four legs are arranged at 90° intervals along the same circumference.

The fundamental principle of active vibration isolation and pointing control in the integrated platform involves transmitting pitch and yaw angle measurements from sensors to the controller. The control algorithm then generates driving currents for the actuators, which, in turn, move the legs to achieve vibration isolation and pointing in the pitch and yaw directions. Notably, the driving currents for legs 1 and 3 have equal magnitudes but opposite directions, as do the driving currents for legs 2 and 4. This configuration enables vibration isolation and pointing control in pitch and yaw directions through a push-pull mechanism.

### 2.2. Dynamic Model

**Figure 2:**
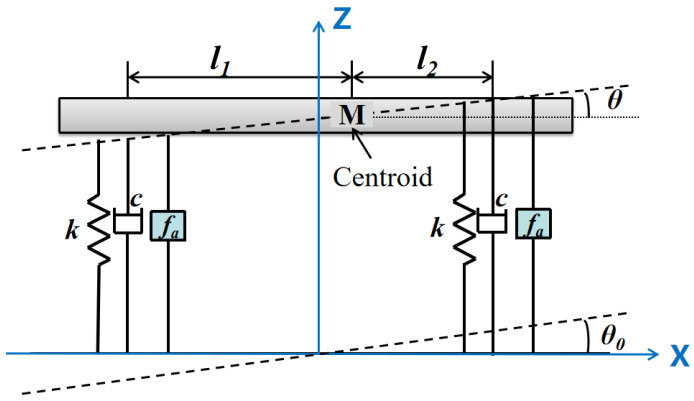
Schematic diagram of the dynamic model.

The integrated vibration isolation and pointing platform has a decoupling design and exhibits symmetry, allowing for its simplification into a two-degree-of-freedom dynamic system as illustrated in Figure 2. The figure depicts rotation around the Y-axis, specifically motion in the yaw direction. k represents the axial stiffness of the elastic element on the leg, c denotes the damping, $f_{a}$ signifies the electromagnetic driving force, $\theta_{0}$ indicates the input disturbance angle, and θ is the rotation angle of the payload.

Let l represent half of the distance between two legs, and define the eccentricity coefficient $e\in \left(0,1\right)$ as the ratio between the distance from the centroid to the geometric center and l. Consequently, we have $l_{1}=l+el$ and $l_{2}=l-el$. Based on these parameters, the two-degree-of-freedom dynamic equations are formulated as follows:

(1) $$J\ddot{\theta }=-2k\theta l^{2}\left(1+e^{2}\right)+2k\theta_{0}l^{2}+2kzle-2c\dot{\theta }l^{2}\left(1+e^{2}\right)+2c{\dot{\theta }}_{0}l^{2}+2c\dot{z}le+2lf_{a}$$

(2) $$m\ddot{z}=-2kz-2c\dot{z}+2k\theta el+2c\dot{\theta }el$$

where m represents the mass of the payload, and J denotes the moment of inertia of the payload around the Y-axis.

We define state variables as $y_{1}=\dot{\theta }$, $y_{2}=\dot{z}$, $y_{3}=\left(1+e^{2}\right)\theta -\theta_{0}$, $y_{4}=z$, and $y_{5}=\theta$. Then, the equations above can be represented in state space form as follows:

(3) $$\left\{\begin{array}{c} \dot{Y}=AY+BU\\ Z=CY+DU \end{array}\right.$$

here,

$$Y=\left[\begin{array}{c} y_{1}\\ y_{2}\\ y_{3}\\ y_{4}\\ y_{5} \end{array}\right]=\left[\begin{array}{c} \dot{\theta }\\ \dot{z}\\ \left(1+e^{2}\right)\theta -\theta_{0}\\ z\\ \theta \end{array}\right],\;U=\left[\begin{array}{c} u_{1}\\ u_{2} \end{array}\right]=\left[\begin{array}{c} {\dot{\theta }}_{0}\\ f_{a} \end{array}\right]$$

$$A=\left[\begin{array}{ccccc} \frac{-2cl^{2}\left(1+e^{2}\right)}{J} & \frac{2cle}{J} & \frac{-2kl^{2}}{J} & \frac{2kle}{J} & 0\\ \frac{2cle}{m} & \frac{-2c}{m} & 0 & \frac{-2k}{m} & \frac{2kel}{m}\\ 1+e^{2} & 0 & 0 & 0 & 0\\ 0 & 1 & 0 & 0 & 0\\ 1 & 0 & 0 & 0 & 0 \end{array}\right],\;B=\left[\begin{array}{cc} \frac{2cl^{2}}{J} & \frac{2l}{J}\\ 0 & 0\\ -1 & 0\\ 0 & 0\\ 0 & 0 \end{array}\right]$$

$$C=\left[\begin{array}{ccccc} 1 & 0 & 0 & 0 & 0\\ 0 & 1 & 0 & 0 & 0\\ 0 & 0 & 1 & 0 & 0\\ 0 & 0 & 0 & 1 & 0\\ 0 & 0 & 0 & 0 & 1 \end{array}\right],\;D=\left[\begin{array}{ccccc} 0 & 0 & 0 & 0 & 0\\ 0 & 0 & 0 & 0 & 0\\ 0 & 0 & 0 & 0 & 0\\ 0 & 0 & 0 & 0 & 0\\ 0 & 0 & 0 & 0 & 0 \end{array}\right]$$

### 2.3. Controller Design

This paper employs a PID controller to achieve vibration isolation and pointing control. The mathematical model of the PID controller is expressed as follows:

(4) $$u\left(t\right)=K_{p}e\left(t\right)+K_{i}\int e\left(t\right)d\left(t\right)+K_{d}\frac{de\left(t\right)}{d\left(t\right)}$$

here, $u\left(t\right)$ represents the controller output; $e\left(t\right)$ denotes the controller input, which signifies the difference between the reference target value and the actual output value of the controlled mechanism; and $K_{p}$, $K_{i}$, and $K_{d}$ correspond to the proportional gain, integral gain, and differential gain of the controller, respectively.

Subsequently, the expression for the active force can be formulated as follows:

(5) $$f_{a}=-K_{p}\left[\theta \left(t\right)-\theta_{r}\left(t\right)\right]-K_{i}\int \left[\theta \left(t\right)-\theta_{r}\left(t\right)\right]d\left(t\right)-K_{d}\frac{d\left[\theta \left(t\right)-\theta_{r}\left(t\right)\right]}{d\left(t\right)}$$

where $\theta_{r}\left(t\right)$ represents the reference target value.

### 2.4. Analysis

#### 2.4.1. Vibration Isolation Analysis

In the process of payload vibration isolation, the reference target value is set to zero. By substituting Equation (5) into Equation (1) and subsequently applying Laplace transformation to Equations (1) and (2), we can derive the transfer function. This function represents the relationship between the base disturbance angle and the payload rotation angle under active vibration isolation control, expressed as follows:

(6) $$\frac{\theta \left(s\right)}{\theta_{0}\left(s\right)}=\frac{\left(ms^{2}+2k+2cs\right)\left(2cl^{2}s^{2}+2kl^{2}s\right)}{\left(ms^{2}+2k+2cs\right)\left[Js^{3}+2cl^{2}s^{2}\left(1+e^{2}\right)+2kl^{2}s\left(1+e^{2}\right)+2l\left(K_{p}s+K_{i}+K_{d}s^{2}\right)\right]-{\left(2kle+2csle\right)}^{2}}$$

**Figure 3:**
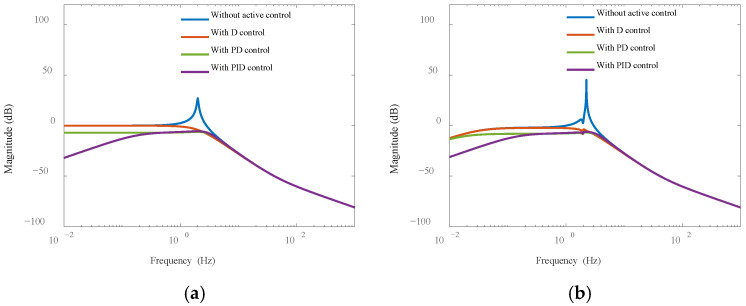
Amplitude-frequency characteristics of closed-loop transmission: (**a**) *e* = 0; (**b**) *e* = 0.5.

The system parameters of the model are: m = 260 kg, k = 20,000 N/m, c = 70 N·s/m, J = 40.49 kg·m^2^. Figure 3 illustrates the closed-loop transfer amplitude–frequency characteristic diagrams obtained using the differential (D), proportional–derivative (PD), and PID controllers under e=0 and e=0.5. The rotational frequency of the vibration isolation system is low, specifically at 2 Hz, due to the small stiffness and large moment of inertia of the payload. Consequently, without active control, the vibration isolation system functions as a passive system capable of suppressing high-frequency vibrations. However, a relatively large peak occurs at the fundamental frequency. The D controller reduces amplitudes around the fundamental frequency, demonstrating the effect of active damping force. The PD controller not only exhibits a damping effect but also increases stiffness, improving control over low-frequency disturbances. The PID controller more effectively reduces amplitude at low frequencies. When the eccentricity coefficient is e=0.5 (Figure 3b), the system frequency changes to 2.25 Hz, but the control effect remains largely unaffected, indicating that the deviation of the center of mass from the geometric center does not significantly impact the control effect in closed-loop vibration isolation.

#### 2.4.2. Pointing Analysis

**Figure 4:**
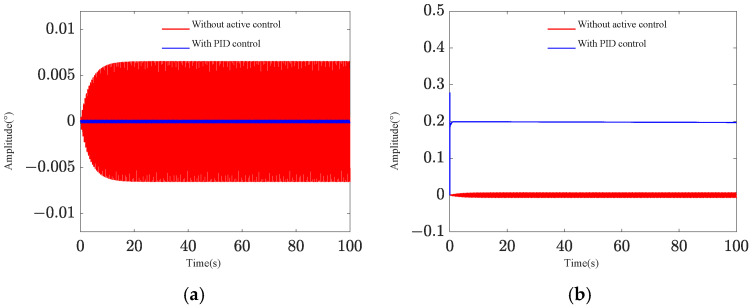
Fixed-point pointing effect: (**a**) 0°; (**b**) 0.2°.

**Figure 5:**
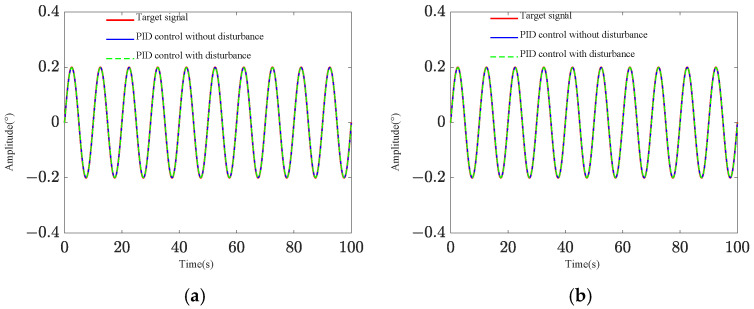
Sinusoidal pointing effect: (**a**) *e* = 0; (**b**) *e* = 0.5.

**Figure 6:**
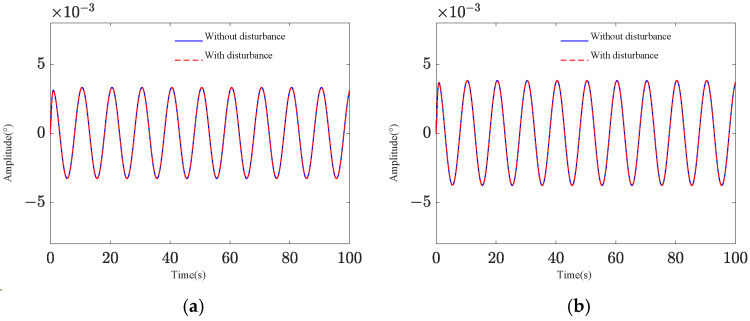
Sinusoidal pointing error: (**a**) *e* = 0; (**b**) *e* = 0.5.

**Figure 7:**
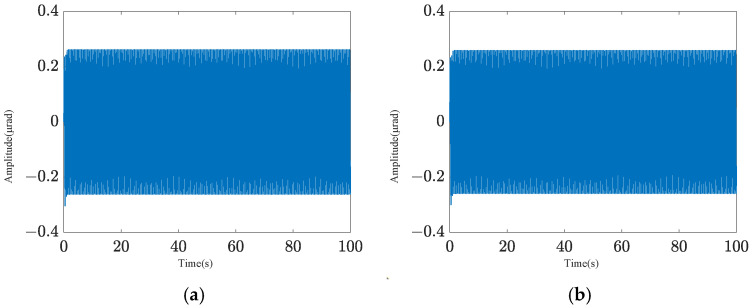
Target difference of PID control with and without disturbance: (**a**) sinusoidal pointing (*e* = 0); (**b**) sinusoidal pointing (*e* = 0.5).

Two pointing scenarios are analyzed: fixed-point pointing at 0° and 0.2°, and sinusoidal tracking pointing at 0.2 × sin (0.2πt)° under 0.1 Hz. A sinusoidal disturbance signal of 5 × sin (4πt) μrad at the fundamental frequency is applied to the base, and the tracking and pointing effects of both scenarios under the disturbance condition are examined (Figure 4 and Figure 5). The results demonstrate that the platform can achieve target tracking with and without disturbance using PID control. The maximum errors of sinusoidal pointing are 1.65% (Figure 6a) and 1.90% (Figure 6b) under e=0 and e=0.5, respectively. The reason why the difference between sinusoidal pointing control with and without disturbances cannot be seen in the curve is mainly because the amplitude of the tracked signal differs too much from the amplitude of the response caused by the disturbance. The disturbance amplitude is 5 µrad, and the response amplitude caused by the disturbance is approximately 0.25 µrad under PID control, as shown in Figure 7. However, the amplitude of the tracked signal is 0.2°, which is 3490 µrad. They are not on the same scale in the image, so it will have a small impact on the tracking results from the curve.

## 3. Integrated Platform Design

The technical specifications for this two-dimensional integrated vibration isolation and pointing platform are as follows. The anticipated fundamental frequency is 2 Hz, and the pointing adjustment range is ±0.2°. Table 1 presents the model parameters simulating the optical payload.

The legs play a crucial role in accomplishing the vibration isolation and pointing objectives. To achieve tracking and pointing capabilities, voice coil motors will serve as the primary actuating elements. The membrane is engineered to function as an elastic component, facilitating passive vibration isolation. Consequently, the leg design encompasses the selection of voice coil motors, membrane design, and flexible joint design. The voice coil motor and membrane will be integrated to form a cohesive actuator unit.

### 3.1. Membrane

In the scenario where the centroid is positioned at the geometric center and $J=J_{x}=J_{y}$, the dynamic equations for yaw and pitch directions can be articulated as follows:

(7) $$J\ddot{\theta }+2cr^{2}(\dot{\theta }-{\dot{\theta }}_{0})+2kr^{2}(\theta -\theta_{0})=2rf_{a}$$

The designed platform exhibits a fundamental frequency of 2 Hz, as determined by the following equation:

(8) $$\frac{\sqrt{\frac{2kr^{2}}{J}}}{2\pi }=2\;\mathrm{Hz}$$

Consequently, the aggregate membrane stiffness on a single leg is calculated as k= 19,979.53 N/m.

**Figure 8:**
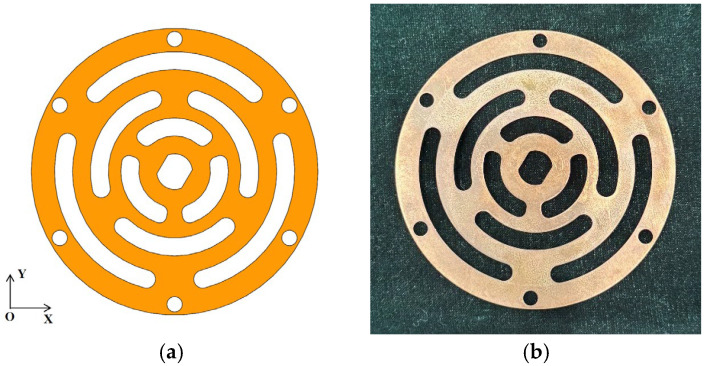
Membrane: (**a**) model; (**b**) real object.

To achieve relatively low axial stiffness and high radial stiffness in the legs while preventing collisions and friction between the coils and magnetic steel in the voice coil motor, a double membrane design is implemented for the actuator. The stiffness of a single membrane is approximately 10,000 N/m. In order to ensure low axial stiffness and minimizing the maximum stress, the membrane with the multi-layer winding pattern in ref. [30] is designed as illustrated in Figure 8 and is manufactured through wire cutting processing. The membrane is made of beryllium bronze, a material chosen for its high strength, hardness, elastic limit, and fatigue resistance. Additionally, beryllium bronze exhibits minimal elastic hysteresis and remains unaffected by magnetic fields.

**Figure 9:**
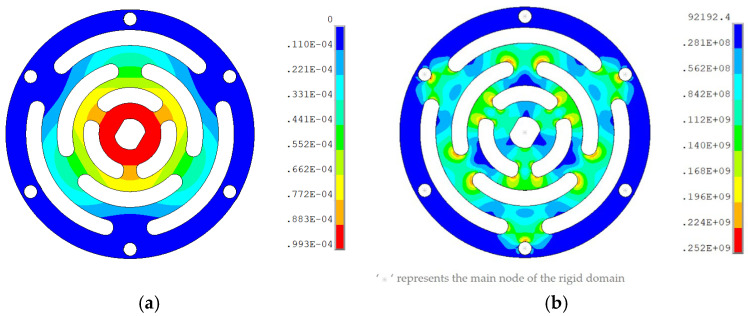
Results of finite element analysis: (**a**) displacement nephogram; (**b**) stress nephogram.

The membrane thickness, determined through finite element analysis, is 0.48 mm. Figure 9a illustrates the membrane deformation when a 1 N load is applied at its center. For a payload rotation angle of 0.2°, the membrane displacement should reach 1.4 mm. To evaluate the membrane’s performance, stress analysis is conducted at the fixed support boundary with an axial displacement of 1.4 mm (Figure 9b). The results indicate that at a payload rotation angle of 0.2°, the maximum stress on the membrane is 252 MPa, occurring at the small arc of the membrane pattern. This stress value remains below the material’s yield stress of 1105 MPa.

### 3.2. Voice Coil Motors

Voice coil motors possess several advantages, including a simple structure, compact size, high precision, linear motion capability, and rapid response. These attributes have led to their widespread adoption across various fields. The operational principle of voice coil motors is based on the generation of electromagnetic force when an energized coil is placed within a magnetic field, enabling swift linear motion. However, the performance of voice coil motors is primarily limited by their travel range and output force range. To meet specific technical requirements, it is therefore essential to select an appropriate voice coil motor for the intended application.

The payload has a radius of 0.4 m, and the voice coil motor should have a displacement of ±1.40 mm to meet the ±0.2° rotation requirement of the platform. Consequently, the total output displacement of the voice coil motor needs to be at least 2.80 mm. Additionally, the relationship between the driving force and the rotation angle is described by the following transfer function:

(9) $$\frac{\theta }{f_{a}}=\frac{2r}{\left(Js^{2}+2cr^{2}s+2kr^{2}\right)}$$

Table 2 presents the calculated output forces necessary to fulfill the index requirements at various frequencies. To meet the technical specification of a ±0.2° rotation angle, the voice coil motor must generate an output force exceeding 28 N.

**Figure 10:**
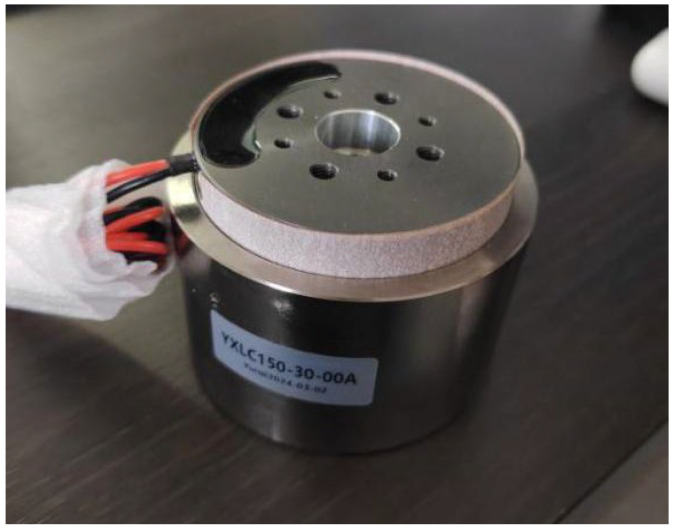
Voice coil motor.

Based on the requisite output displacement and force, the YXLC150-30-00A voice coil motor (Figure 10) is selected. The performance parameters of this motor are presented in Table 3.

### 3.3. Flexible Joint

**Figure 11:**
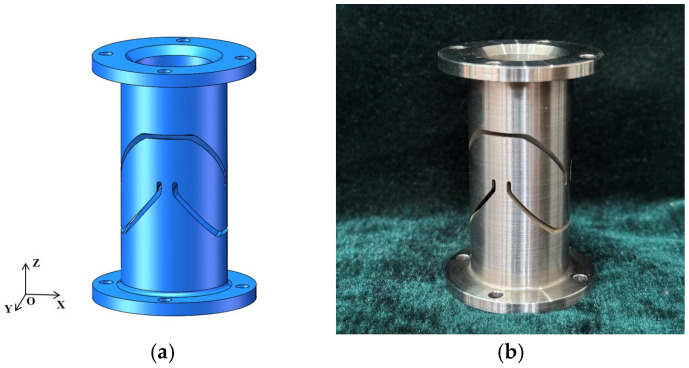
Flexible joint: (**a**) model; (**b**) real object.

**Figure 12:**
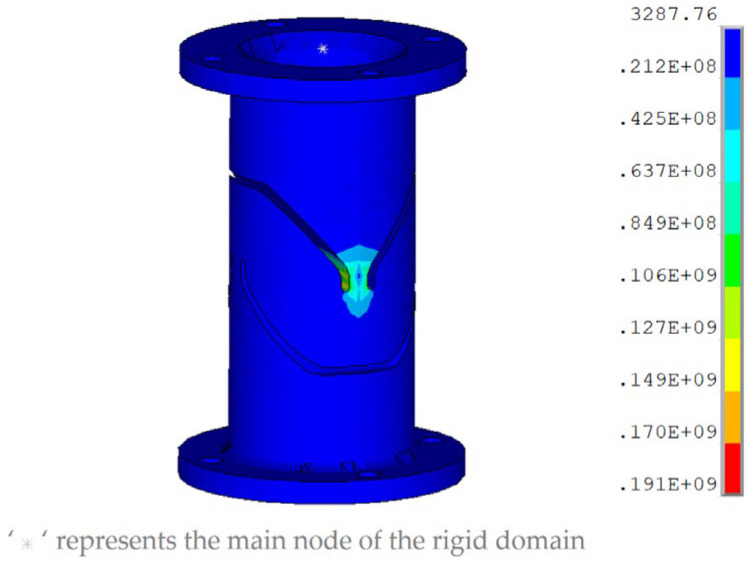
Stress nephogram of the flexible joint.

The joint is a crucial component that connects the legs with the payload and lower platform, facilitating flexible payload movement. To achieve seamless, frictionless, and high-precision rotation of the payload, the flexible joint in ref. [30] is employed, as shown in Figure 11. Steel is selected as the material, and the internal channels are processed by wire-cutting with slow-traveling wire electrical discharge machining. When the pitching or yawing angle of the platform reaches 0.2°, the bending moment acting on the X-axis of the flexible joint is approximately 2.6 N·m. The bending stress of the flexible joint is analyzed using the finite element method. Initially, a fixed constraint is applied at one end of the flexible joint. A key point is established at the center of the other end of the joint, and a rigid domain is created. Bending moments with a magnitude of 2.6 N·m are applied to the key point. Figure 12 demonstrates the finite-element analysis results. The analysis reveals that the maximum stress of the flexible hinge appears at the center of the joint, with a value of 191 MPa, indicating that the bending deformation remains within the purely elastic range.

### 3.4. Actuator

**Figure 13:**
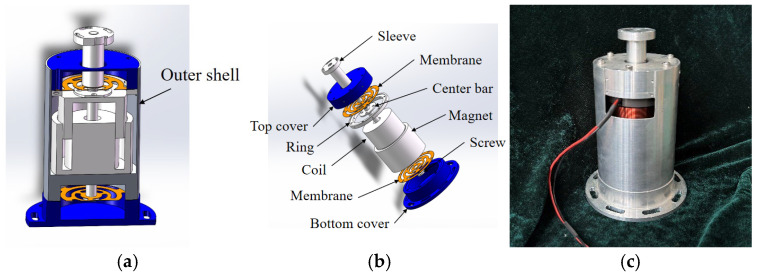
Actuator: (**a**) cross-sectional view; (**b**) structural components; (**c**) real object.

Figure 13a,b present the three-dimensional (3D) schematic diagram of the designed actuator, while Figure 13c displays a photograph of the actual device. The actuator’s central bar is attached to the upper and lower membranes, enabling low axial stiffness and comparatively high radial stiffness. This design allows for axial movement of the coils while preventing collisions and friction with the permanent magnets. The sleeve and coil, also connected to the central bar, constitute the moving components. The stationary elements, including the top cover, ring, magnet, outer shell, and bottom cover, are securely fastened in sequence, with the bottom cover firmly attached to the base.

The operational mechanism of this actuator is as follows: External disturbances acting on the bottom cover transmit force to the upper and lower membranes, inducing their vibrations. High-frequency vibrations are passively isolated due to the membrane’s low axial stiffness, while low-frequency vibrations pass through. The vibrations of the membranes drive the central bar to oscillate, which, in turn, causes the connected coil to vibrate. The coil’s movement within a magnetic field generates a damping effect. Concurrently, the payload’s rotation signal, measured by a sensor, is transmitted to the controller. The controller then calculates the appropriate control current, which drives the voice coil motor to produce the corresponding displacement, thereby achieving active vibration isolation during the pointing process.

### 3.5. Leg

**Figure 14:**
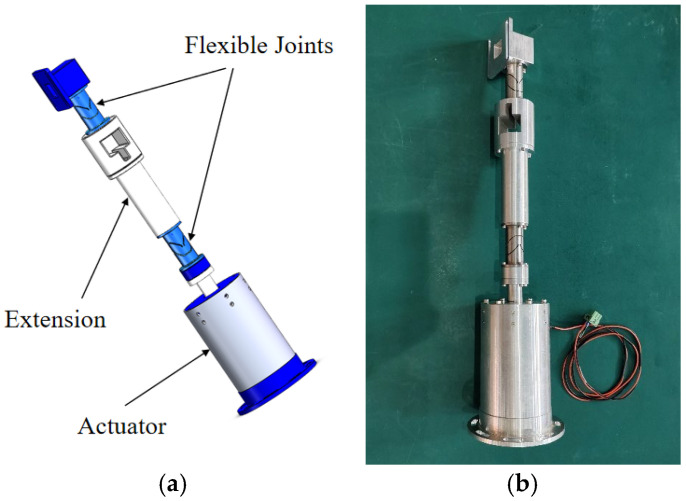
Single-leg: (**a**) model; (**b**) real object.

The model and photograph of the single-leg configuration are presented in Figure 14. The upper flexible joint is connected to the payload via a square adapter. An extension bar is positioned between the two flexible joints. The lower flexible joint is attached to the actuator through a connecting piece. The leg’s lower end is affixed to the platform’s base.

### 3.6. Platform

**Figure 15:**
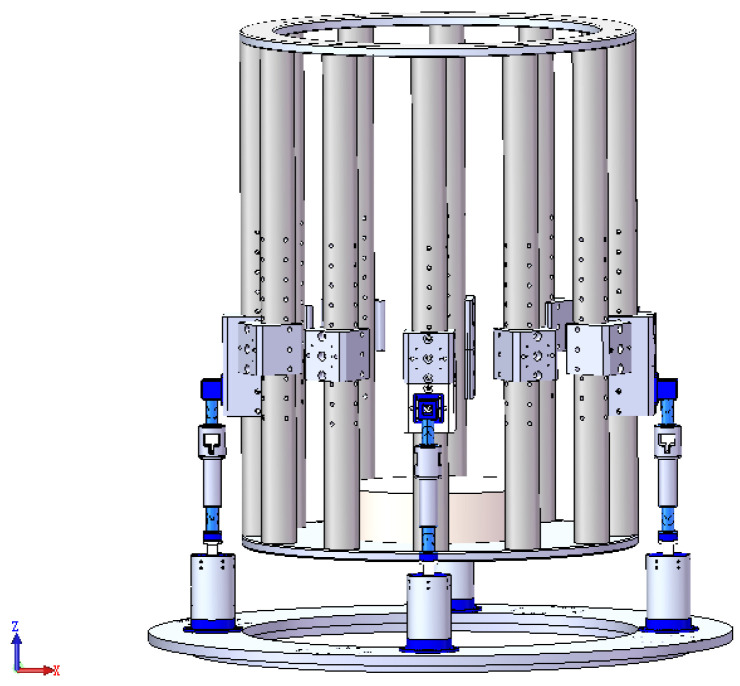
Integrated platform model for vibration isolation and pointing.

The integrated vibration isolation and pointing platform is constructed by assembling the payload, four legs, and base. Figure 15 illustrates a 3D diagram of the designed integrated vibration isolation and pointing platform with its payload.

## 4. Experiment Section

### 4.1. Experimental Setup

**Figure 16:**
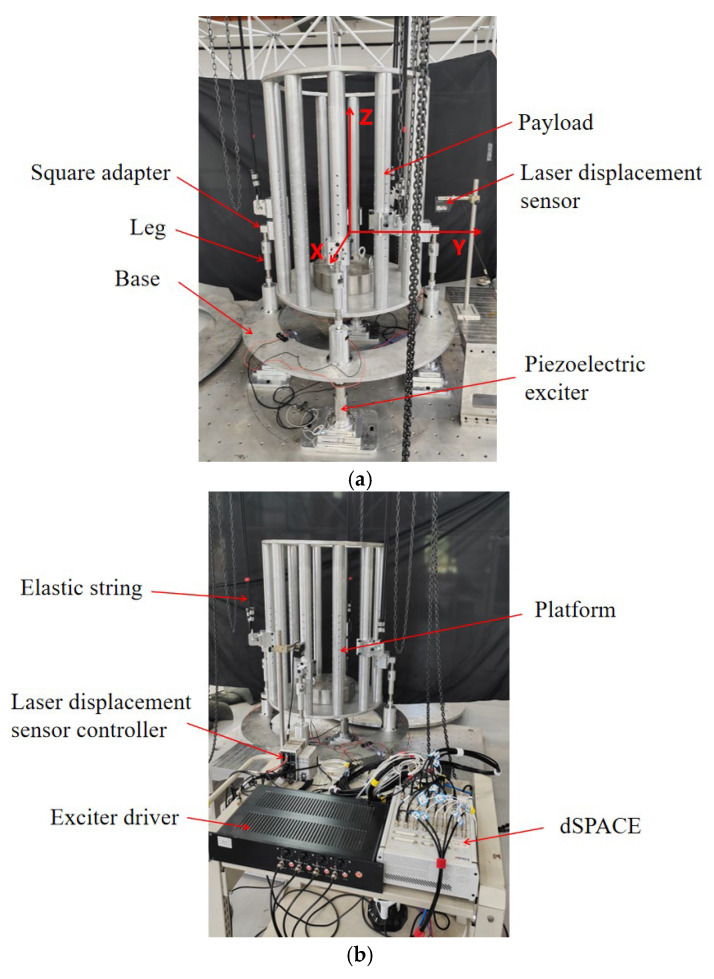
Ground model of the integrated micro-vibration isolation and pointing platform: (**a**) on-ground testing platform; (**b**) connection between platform and device.

Figure 16 illustrates the experimental setup for this integrated vibration isolation and pointing platform. To mitigate gravitational effects, the payload model is suspended using elastic strings. The gravity unloading frequency is approximately 0.4 Hz, which is 1/5 of the fundamental frequency. Four high-thrust piezoelectric actuators, installed at the platform base’s bottom, serve as the micro-vibration simulation disturbance source. A laser displacement sensor indirectly measures the payload rotation angle, which is calculated using the measured displacement and the load’s radius. The control algorithm is developed using MATLAB/Simulink R2023b and subsequently compiled and evaluated using dSPACE ControlDesk 2020 to assess control effectiveness. Concurrently, dSPACE facilitates sensor signal acquisition and voltage signal transmission. The custom-designed current drive primarily converts voltage signals into current signals to drive the actuator. Given the symmetry of motion in the pitch and yaw directions, the vibration isolation and pointing control are verified using pitch direction movement as an example in the ground test, where the load rotates around the X-axis, as shown in Figure 16a.

### 4.2. Experimental Results

**Figure 17:**
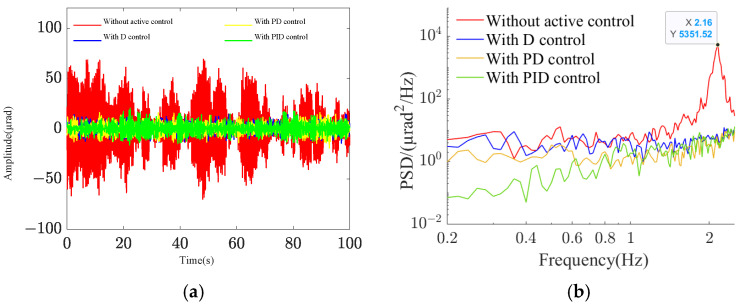
Low-frequency control effects under different control schemes: (**a**) time-domain response; (**b**) power spectral density.

**Figure 18:**
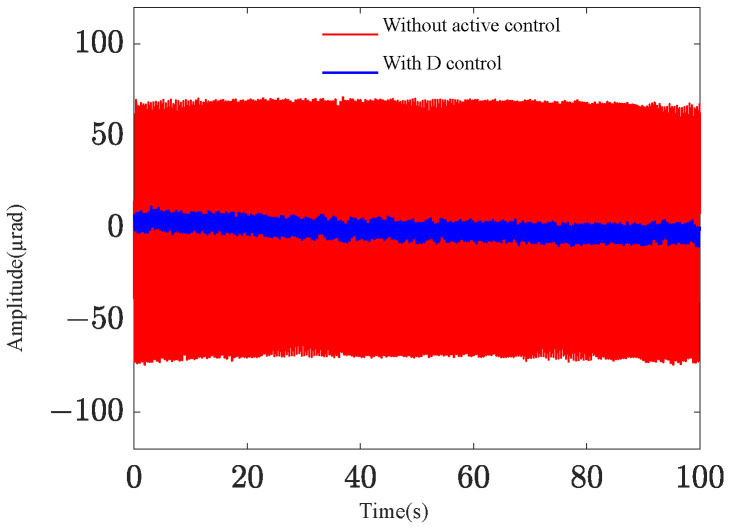
D control effect at the fundamental frequency.

To evaluate the effectiveness of active vibration control at low frequencies, a random excitation with a frequency bandwidth of 0.2 Hz to 2.5 Hz and a root mean square value of 8.65 µrad is applied to the base. Figure 17 illustrates the vibration control effects under different controllers. The integrated platform’s fundamental frequency is observed to be 2.16 Hz, aligning with the expected design value. Analysis of the time-domain response and power spectral density graphs reveals that the payload’s response amplitude can be effectively controlled. Notably, the PID control demonstrates superior control at low frequencies, consistent with theoretical analysis predictions. Figure 18 depicts the D control effect at the fundamental frequency. The pitch–yaw angle response at the mass center plane decreases from approximately 70 µrad without active control to about 5 µrad, representing a reduction of approximately 23 dB.

**Figure 19:**
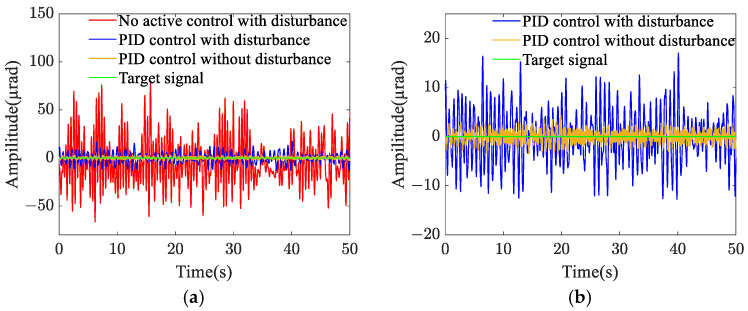
Control effect of fixed-point pointing at 0°: (**a**) comparison of control effects; (**b**) PID control effect without disturbance.

**Figure 20:**
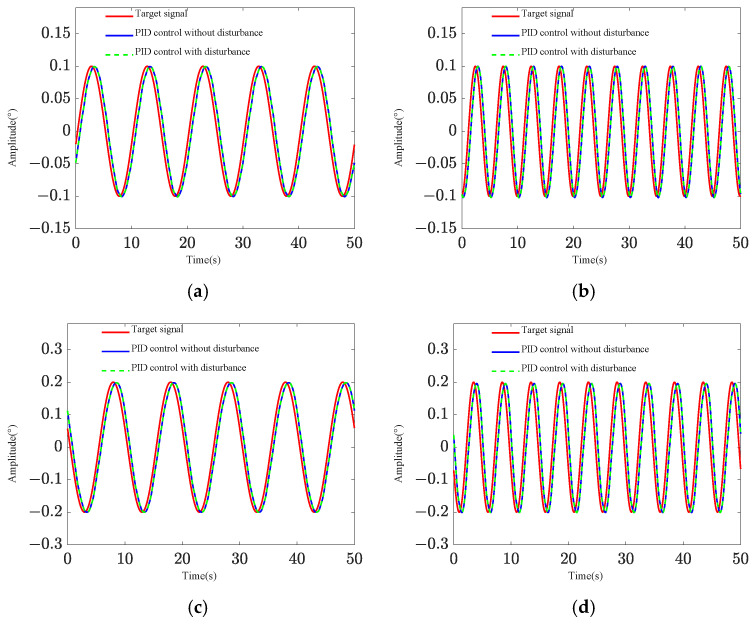
Tracking performance under varied pointing signals: (**a**) amplitude: 0.1°; frequency: 0.1 Hz; (**b**) amplitude: 0.1°; frequency: 0.2 Hz; (**c**) amplitude: 0.2°; frequency: 0.1 Hz; (**d**) amplitude: 0.2°; frequency: 0.2 Hz.

The efficacy of fixed-point pointing control at 0° is illustrated in Figure 19. When subjected to random base disturbances, the root-mean-square (RMS) value of the pitch angle response amplitude is 24.80 µrad without active control and 5.14 µrad under PID control. In the absence of disturbances, the vibration angle is maintained within 1.20 µrad. Figure 20 demonstrates the tracking performance following the input of sinusoidal pointing signals with amplitudes of 0.1° and 0.2° at frequencies of 0.1 Hz and 0.2 Hz, respectively. The integrated platform exhibits successful pointing tracking of the signals with and without disturbance. However, due to filtering in actual control systems, phase differences may occur in tracking and pointing control for space loads with significant mass. As the tracking frequency increases, the phase difference rises. Table 4 presents the phase differences under various sinusoidal pointing conditions. Future research will focus on optimizing the control algorithm to mitigate these phase differences.

## 5. Conclusions

This paper presents the design of an integrated micro-vibration isolation and pointing platform featuring a four-leg structure with pitch and yaw adjustment capabilities utilizing voice coil motors. The platform comprises three primary components: a payload, legs, and a base. The legs incorporate voice coil motors, membranes, and flexible joints as their essential elements. Our research yields several significant findings:

(1) Altering the position of the payload deviation affects the system’s fundamental frequency; however, it exerts minimal influence on the control effectiveness.

(2) The D controller demonstrates the impact of active damping force. The PD controller exhibits damping and stiffness effects, enhancing control over low-frequency disturbances. In contrast, the PID controller more effectively reduces the amplitude at low frequencies.

(3) The design of the upper and lower membranes facilitates low axial stiffness while maintaining comparatively high radial stiffness. This configuration enables axial movement of the coils while preventing collisions and friction with the permanent magnets.

(4) The integrated micro-vibration isolation and pointing platform demonstrates an attenuation effect of 23 dB at the fundamental frequency. Under random excitation conditions, the RMS value of the angular response amplitude is 24.80 µrad without active control and 5.14 µrad with PID control.

(5) The integrated platform demonstrates the capability to track and point at signals in the presence and absence of disturbances. However, phase differences are observed during the tracking and pointing process for space loads with a significant mass. Future research will focus on optimizing the control algorithm to mitigate these phase differences. Furthermore, achieving a wider pointing angle remains a key objective of our ongoing investigations.

## Figures and Tables

**Table 1:** Parameters of the optical payload model.

Parameter	Value
Mass	260 kg
Platform radius	0.40 m
$\mathrm{Moment}\;\mathrm{of}\;\mathrm{inertia}\;(J_{x}$)	40.49 kg·m^2^
$\mathrm{Moment}\;\mathrm{of}\;\mathrm{inertia}\;(J_{y}$)	40.49 kg·m^2^
$\mathrm{Moment}\;\mathrm{of}\;\mathrm{inertia}\;(J_{z}$)	22.26 kg·m^2^

**Table 2:** Output force required by the voice coil motor at different frequencies.

Frequency (Hz)	Output Force (N)
0.1	28
0.2	28
0.3	27.5
0.4	26.8
0.5	26.2

**Table 3:** Performance parameters of the YXLC150-30-00A voice coil motor.

Parameter	Value
Peak force	150 N
Continuous force	40.8 N
Force constant	19.5 N/A
Stroke	30 mm
Mass of the coil assembly	330 g
Mass of the magnet assembly	1170 g

**Table 4:** Phase differences under different sinusoidal pointing conditions.

Pointing Condition	Phase Difference
Amplitude: 0.1°; Frequency: 0.1 Hz	5.26%
Amplitude: 0.1°; Frequency: 0.2 Hz	8.81%
Amplitude: 0.2°; Frequency: 0.1 Hz	5.44%
Amplitude: 0.2°; Frequency: 0.2 Hz	10.81%

## Data Availability

Data are contained within the article.

## Abbreviations

The following abbreviations are used in this manuscript:

PID	proportional–integral–derivative
HST	Hubble Space Telescope
JWST	James Webb Space Telescope
PPH	Precision Pointing Hexapod
3D	three-dimensional
RMS	root-mean-square
